# Distribution and clinical associations of integrating conjugative elements and *cag* pathogenicity islands of *Helicobacter pylori* in Indonesia

**DOI:** 10.1038/s41598-018-24406-y

**Published:** 2018-04-17

**Authors:** Langgeng Agung Waskito, Muhammad Miftahussurur, Maria Inge Lusida, Ari Fahrial Syam, Rumiko Suzuki, Phawinee Subsomwong, Tomohisa Uchida, Muhammad Hamdan, Nasronudin, Yoshio Yamaoka

**Affiliations:** 1 Department of Environmental and Preventive Medicine, Oita University Faculty of Medicine, Yufu, Japan; 2 Institute of Tropical Disease, Universitas Airlangga, Surabaya, Indonesia; 3 Gastroentero-Hepatology Division, Department of Internal Medicine, Faculty of Medicine, Universitas Airlangga, Surabaya, Indonesia; 4 Division of Gastroenterology, Department of Internal Medicine, Faculty of Medicine, University of Indonesia, Jakarta, Indonesia; 5 Department of Molecular Pathology, Oita University Faculty of Medicine, Yufu, Japan; 6 Faculty of Medicine, Universitas Muhammadiyah Surabaya, Surabaya, Indonesia; 7 Department of Medicine, Gastroenterology and Hepatology section, Baylor College of Medicine, Houston, Texas USA

## Abstract

The clinical associations and correlations with other virulence factors such as *cag* pathogenicity island (PAI) of the Integrating Conjugative Elements *Helicobacter pylori* TFSS (ICE*Hptfs*), a new type IV secretion system (TFSS) in *H. pylori* has not been described. Among 103 studied strains from Indonesia, almost all strains (99.0%) contained *cag* PAI with more than half (55.8%) were intact *cag* PAI. Patients infected with intact *cag* PAI strains showed significantly higher antral activity, inflammation and atrophy as well as corporal inflammation than those with non-intact *cag* PAI strains, confirming the virulence of *cag* PAI. Over half of strains (53.8%) contained ICE*Hptfs*, predominantly consisted of ICE*Hptfs3*-*tfs4a* (42.8%) and ICE*Hptfs3* (16.3%). Although patients infected with ICE*Hptfs*-positive strains had lower *H. pylori* density, those with the complete ICE*Hptfs4b* strains tended to have higher antral activity than the negative one. In combination, patients infected with combination of intact *cag* PAI-ICE*Hptfs*-positive strains had more severe inflammation than those with non-intact *cag* PAI-ICE*Hptfs*-negative, suggesting a possibility of a mutual correlation between these TFSS(s).

## Introduction

*Helicobacter pylori* is a human-specific bacterium, colonizing the stomach of approximately 50% of the modern human population^1^. Infection is associated with several gastro-duodenal pathologies, including chronic gastritis, peptic ulcers, and even gastric cancer in a subset of individuals, depending on the variation of bacterial virulence, host genetics and/or environmental factors^2,3^. *H. pylori* is the most genetically diverse pathogenic bacteria^4,5^, which might be associated with frequent horizontal gene transfer (HGT) and recombination within species as an adaptation process to the host over years of infection^6^. Therefore, its genome contains many putative genes, which are generally classified into three categories^7^. The first is phase-variable genes, defined as those with functional status that could change due to particular conditions. An example is the slipped-strand mispairing mechanism, which often shown in outer membrane proteins such as *oipA* and *sabA*, that can switch genes ‘on’ and ‘off’ very rapidly^8,9^. The second is genes with different structures/genotypes, such as the repeat region of CagA. The 3′ repeat region of CagA can differ between two genotypes, associated with a different risk of developing gastric cancer^10,11^. The last is strain-specific genes, defined if the gene only exists in a particular strain. The most studied genes in this category are *cag* pathogenicity island (PAI), which encodes a type IV secretion system (TFSS)^7,12^.

TFSS is a flexible secretion system found in both Gram-positive and -negative bacteria. In Gram-negative bacteria, it mediates secretion of various protein substrates, from monomeric proteins, multi-subunit protein toxins and nucleoprotein complexes^13^. Importantly, more than one TFSS could be found in one species of bacteria, including in the *H. pylori*. *H. pylori* has four types of TFSS with varied functionalities^4,12,14,15^. The first is *cag* PAI, which primarily injects CagA into host cells^12^. The second is the *comB* system which has a principal function of DNA uptake and natural transformation within *H. pylori* genome^15^. The most recently revealed TFSS is within Integrating Conjugative Elements (ICEs). In case of *H. pylori*, this is known as ICE *H. pylori* TFSS (ICE*Hptfs*)^16^. ICE*Hptfs* was initially named as plasticity regions, the regions within the *H. pylori* genome which have considerably lower guanine and cytosine content (~35%) compared with the rest of the genome (39%)^17^. The lower G + C content indicates that plasticity regions may be the result of HGT^8,17^. With the increasing number of *H. pylori* complete genomes deposited in the GenBank, plasticity regions are considered as conserved mobile elements, rather than a region with genomic plasticity, and are usually organized as a complete set of TFSS machinery. In addition, based on the acquisition of these elements through conjugative HGT, these elements are best described as ICEs. The TFSS within ICE*Hptfs*(s) is called TFSS3 and TFSS4a/4b/4c^4,16^. Those differences between ICE*Hptfs*3 and ICE*Hptfs*4a/b/c were determined by the nucleotide diversity of the *virB-virD* orthologues genes, resulting very distinctive diversity between TFSS3 and TFSS4 in general^16^. In addition, the TFSS4 possesses sub-type based on nucleotide diversity in the *virB2*, *virB3*, *virB4*, *topA*, *virB7* and *virB8*, discriminating TFSS4a and TFSS4b, and diversity in *virB11*, *virD4* and *virD2*, distinguishing TFSS4a and TFSS4c^16^. The terminology of this region was inconsistent in several previous studies. A study conducted in 2009 reported a new TFSS termed as TFSS3, TFSS3a, and TFSS3b^14^ within a mobile element called transposon of plasticity zones (TnPZ) type 2 for TFSS3, TnPZ1 for TFSS3a and TnPZ1b for TFSS3b. However a year later the TFSS3b were termed as TFSS4 for the TFSS inside the mobile element plasticity zones 1 (PZ1) and TFSS3 for the TFSS inside the mobile element PZ3. Following with the newest terminology is ICE*Hptfs*3 which containing TFSS3 and ICE*Hptfs*4a/4b/4c which containing TFSS4a/4b/4c^16^. In order to make consistent terminology, in this study we used ICE*Hptfs3* and ICE*Hptfs4a/4b/4c* for TFSS3 and TFSS4a/4b/4c, respectively^16^. Currently, the distribution and association of these new TFSSs to gastro-duodenal diseases are not fully described.

Indonesia is a country in South-East Asia, consisting of more than 13,600 islands and 400 ethnicities^18^. As described previously, *H. pylori* infected the ancestors of modern humans in Africa about 100,000 years ago (100 kya) and migrated with its host from Africa to Asian and American continents^19,20^. Therefore, ethnic diversity is associated with *H. pylori* infection as well as genome diversity, especially in Indonesia. We have described that ethnicity is a risk factor for *H. pylori* infection^21^. In addition, ethnicity is also a factor for the diversity of virulence genes in *H. pylori*. We have described that different ethnicity had a different genetic polymorphism on the several virulence genes in the nucleotides and amino acids level. For example, strains possessing pre-EPIYA motif of CagA isolated from Batak ethnic showed 6 bp deletion-type pre-EPIYA motif with East Asian-type CagA. The 6 bp deletion-type is unique type among East Asian-type CagA since almost all pre-EPIYA motif types of strains isolated from Japan and Vietnam was reported to have 39 bp deletion-type and 18 bp deletion-type, respectively^22^. Patients infected with this 6 bp deletion-type/East Asian-type CagA strains showed to have lower gastric mucosal histologic scores compared to those with Western-type CagA^23^, although it is well known that East Asian-type CagA had generally more virulent than Western-type CagA. In addition, the predominant type of CagA was also different in each ethnic group.

As the distribution and clinical association of ICE*Hptfs*(s) have not been reported, it is interesting to investigate the distribution and clinical association of these regions as well as the correlation with other virulence genes in relation to the clinical outcome. Here, we reported the distribution of ICE*Hptfs* in Indonesia using high throughput next-generation sequencing technology and revealed that strains from some geographic areas lack this genomic region, and the intactness of this region had an association with clinical outcome.

## Results

### Characteristic of patients and prevalence of ICE*Hptfs* and *cag* PAI

We performed endoscopic examination on 1072 dyspeptic patients in 17 cities in Indonesia from August 2012 to August 2016, and a total of 103 *H. pylori* were isolated from patients (66 male and 37 female; mean age 49.2 ± 13 years; range 24–80 years), comprising 92 patients with gastritis, 10 with peptic ulcer disease (PUD) and 1 with gastric cancer. Among 103 isolates, 75 isolates were from our previous study with information of *cagA* genotypes^24^. Strains originated in Indonesia are shown in Supplementary Figure 1.

**Figure 1:**
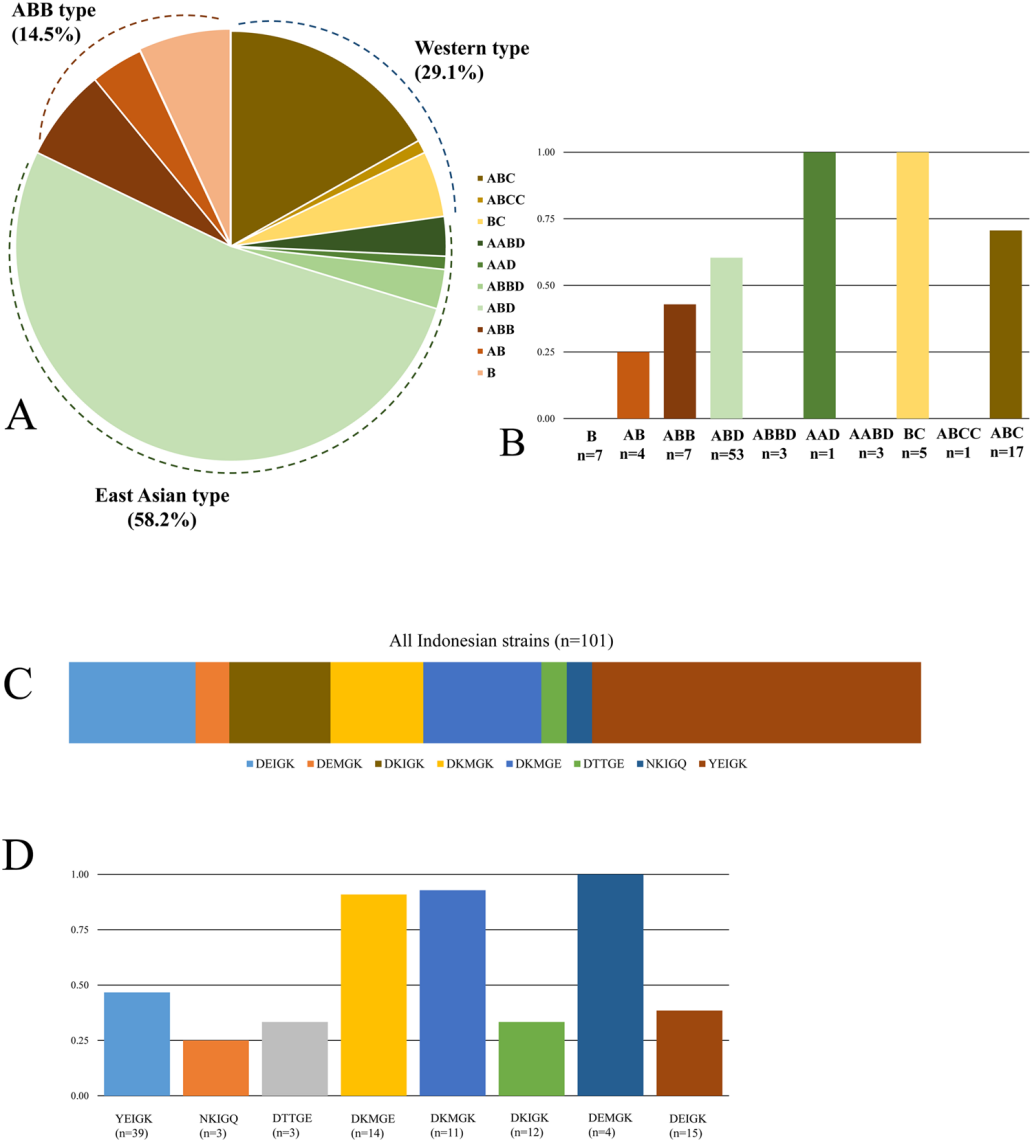
Distribution of CagA and CagL hypervariable motif (CagLHM) and the ICE*Hptfs*. (**A**) The distribution of CagA genotype among Indonesian strains. More than half (58.2%) was East-Asian type CagA. (**B**) The proportion of ICE*Hptfs* among CagA genotype. It showed the B, ABBD, AABD and ABCC type CagA did not possessed ICE*Hptfs*. (**C**) The distribution of CagLHM among Indonesian *cag* PAI positive strains. We observed new CagLHM motif DKMGK. (**D**) The proportion of ICE*Hptfs* observed in the CagLHM motif group showed all NKIGQ motif (n = 3) contained ICE*Hptfs* elements. The new observed DKMGK motif strains showed the high prevalence (10/11, 90.9%) as high as the DKMGE motif strains (13/14, 92.8%), the progenitor motif of CagLHM.

We evaluated the *cag* PAI and determined the functional status of each gene present (Table 1). We categorized these into i) Intact *cag* PAI, if all the genes were detected and there was no deletion, stop codon or frameshift in each gene; ii.) Non-intact *cag* PAI, if at least one of the genes were lacking or had stop codon and/or frameshift in the gene; and iii.) *cag* PAI-negative, if none of the *cag* PAI genes were detected. In total, *cag* PAI was detected in most isolates (99.1%), either intact or non-intact. Among the detected *cag* PAI strains, 57 strains possessed intact *cag* PAI (55.8%). The gastric cancer patient had intact *cag* PAI *H. pylori*. The *cagA* was detected in 101 strains (98%). Sequence analysis of the 27 new *cagA*-positive strains showed that 5 (18.5%) strains possessed Western-type CagA and 12 (44.4%) strains possessed East-Asian type CagA. In addition, we also confirmed a unique genotype of CagA (AB and B type) which mostly were isolated from Merauke city, Papua Island. Those B segment of CagA genotypes had very similar amino acids sequences with ABB type CagA from our previous report^23^ (Supplementary Figure 2). Therefore, we deemed it a subtype of the ABB type CagA. Taken together with our previous study^24^, the result was 60 (58.2%) strains possessed the East-Asian type CagA (AABD, AAD and ABD type), whereas 30 strains (29.1%) were Western-type CagA (ABC, ABCC and BC type) and 15 strains (14.5%) were ABB type CagA (ABB, AB and B) (Fig. 1A).

**Table 1:** Prevalence of ICE*Hptfs* and *cag* PAI.

Characteristic	Total (n = 103)	Clinical Outcome (%)		
		Gastritis (n = 92)	PUD (n = 10)	Cancer (n = 1)
*cagA* positive	101 (98.0)	90 (97.8)	10 (100)	1 (100)
Intact *cag* PAI	57 (55.4)	50 (54.3)	6 (60.0)	1 (100)
non-intact *cag* PAI	45 (43.7)	41 (44.5)	4 (40.0)	0 (0.0)
*cag* PAI negative	1 (0.9)	1 (1.1)	0 (0.0)	0 (0.0)
ICE*Hptfs*	56 (54.3)	51 (55.4)	5 (50.0)	0 (0.0)
**Type of ICE** ***Hptfs***				
TFSS3	9 (16.0)	9 (9.7)	0 (0.0)	0 (0.0)
TFSS4a	8 (14.4)	6 (6.5)	2 (20.0)	0 (0.0)
TFSS4b	6 (10.7)	6 (6.5)	0 (0.0)	0 (0.0)
TFSS3-TFSS4a	24 (42.8)	22 (23.9)	2 (20.0)	0 (0.0)
TFSS3-TFSS4b	5 (8.9)	4 (4.3)	1 (10.0)	0 (0.0)
TFSS3-TFSS4a/b	4 (7.2)	4 (4.3)	0 (0.0)	0 (0.0)

Abbreviations: PAI, patogenicity island; TFSS, type IV secretion system; PUD, peptic ulcer disease.

ICE*Hptfs* were detected in 56 of 103 (54.3%) strains. Among gastritis patients, 51 strains (55.4%) and 5 strains (50.0%) from PUD patients possessed ICE*Hptfs*. Interestingly, the strain isolated from the gastric cancer patient did not contain ICE*Hptfs* (Table 1). Sequence analysis showed there were no mutations leading to premature stop codons or frameshift mutation; thus, we concluded that all of the genes were functional. Among the ICE*Hptfs*-positive strains the single ICE*Hptfs* was observed as ICE*Hptfs3* (16.0%), ICE*Hptfs4a* (14.4%) and ICE*Hptfs4b* (10.7%). There was no strain with ICE*Hptfs4c*. Aside from single ICE*Hptfs* in the genome, strains possessing multiple ICE*Hptfs* were also observed: ICE*Hptfs3-tfs4a* (42.8%), ICE*Hptfs3*-*tfs4b* (8.9%) and ICE*Hptfs3-4a/4b* (7.2%) (Table 1).

### The distribution of ICE*Hptfs* and the ethnic groups

There was a significant association between ethnic group and prevalence of ICE*Hptfs* (P = 0.031). Timor tribe strains had the highest prevalence of ICE*Hptfs* (10/12, 83.3%) and the lowest prevalence was observed in Minahasanese strains (14.2%) (Table 2). There was also a significant association between ethnic groups and the type of ICE*Hptfs* (P = 0.002). Batak tribes possessed predominantly ICE*Hptfs3-tfs4a* (77.8%), whereas Chinese ethnicities possessed predominantly ICE*Hptfs3* (57.1%). As for the Timor ethnicity, the types of ICE*Hptfs* were distributed evenly (Table 2).

**Table 2:** Distribution of *cag* PAI and ICE*Hptfs* among Ethnic Group.

Genetic Profiles	Total (n = 103)	Ethnic Groups (%)								
		Javanese (n = 3)	Chinese (n = 9)	Balinese (n = 6)	Bugis (n = 13)	Batak (n = 31)	Papuan (n = 20)	Minahasanese (n = 7)	Dayak (n = 2)	Timor (n = 12)
ICEHptfs*	56 (54.3)	1 (33.3)	7 (77.8)	4 (66.7)	7 (53.8)	19 (58.0)	6 (30.0)	1 (14.2)	1 (50.0)	10 (83.3)
**Type of ICE** ***Hptfs*** *										
TFSS3	9 (16.3)	0 (0.0)	4 (57.1)	1 (25.0)	2 (28.5)	2 (11.1)	0 (0.0)	0 (0.0)	1 (100)	0 (0.0)
TFSS4a	8 (14.5)	0 (0.0)	1 (14.2)	2 (50.0)	2 (28.5)	2 (11.1)	0 (0.0)	1 (100)	0 (0.0)	0 (0.0)
TFSS4b	6 (10.9)	1 (100)	0 (0.0)	0 (0.0)	1 (14.2)	1 (5.5)	1 (16.7)	0 (0.0)	0 (0.0)	2 (20.0)
TFSS3-TFSS4a	24 (43.6)	0 (0.0)	1 (14.2)	1 (50.0)	2 (28.5)	14 (77.8)	3 (50.0)	0 (0.0)	0 (0.0)	3 (30.0)
TFSS3-TFSS4b	4 (7.2)	0 (0.0)	1 (14.2)	0 (0.0)	0 (0.0)	0 (0.0)	1 (16.7)	0 (0.0)	0 (0.0)	2 (20.0)
TFSS3-TFSS4a/b	4 (7.2)	0 (0.0)	0 (0.0)	0 (0.0)	0 (0.0)	0 (0.0)	1 (16.7)	0 (0.0)	0 (0.0)	3 (30.0)
Complete tfss*										
Incomplete TFSS	32 (57.1)	1 (33.3)	5 (71.4)	3 (75.0)	6 (85.7)	9 (47.3)	4 (66.7)	1 (100)	1 (100)	2 (20.0)
Complete TFSS3	19 (33.9)	0 (0.0)	2 (28.6)	1 (25.0)	0 (0.0)	9 (47.3)	2 (33.3)	0 (0.0)	0 (0.0)	5 (50.0)
Complete TFSS4b	5 (9.0)	0 (0.0)	0 (0.0)	0 (0.0)	1 (14.3)	1 (5.4)	0 (0.0)	0 (0.0)	0 (0.0)	3 (30.0)
***cag*** **PAI**										
*cag* PAI positive	101 (98.0)	3 (100)	9 (100)	6 (100)	12 (92.3)	31 (100)	20 (100)	7 (100)	2 (100)	12 (100)
Intact *cag* PAI	57 (55.3)	0 (0.0)	3 (33.3)	5 (83.3)	8 (72.7)	16 (51.6)	11 (55.0)	2 (28.5)	2 (100)	10 (83.3)

Abbreviations: TFSS, type IV secretion system; PAI, pathogenicity island.

(*) P < 0.05, Fischer’s exact test.

Complete ICE*Hptfs* were assessed as a cluster with complete TFSS machineries, composed of VirB2, VirB3, VirB4, VirB6, VirB7, VirB8, VirB9, VirB9, VirB10, VirB11, VirD2, VirD4, XerT and TopA. Among the positive ICE*Hptfs* strains, 32 strains (57.1%) possessed incomplete ICE*Hptfs*. The complete ICE*Hptfs* were found in 24 strains: 19 (33.9%) with complete ICE*Hptfs3* and 5 (9.0%) with complete ICE*Hptfs4b*. There was a significant association between the completeness of ICE*Hptfs* and the ethnic groups (P = 0.03). Timor tribe strains showed the highest prevalence of complete ICE*Hptfs* (80.0%), in which 5 strains (50.0%) possessed complete ICE*Hptfs3* and 3 strains (30.0%) possessed complete ICE*Hptfs4b* (Table 2).

### The ICE*Hptfs*, CagA and CagL

Among totally 30 strains with Western-type CagA, we could find 18 strains (60.0%) containing ICE*Hptfs* (Supplementary Table 2). However, we could not obtain any ICE*Hptfs* elements from strains which possessed ABCC- and B-type CagA (Fig. 1B). Interestingly, the B-type CagA strains were isolated from Merauke city, Papua island, suggesting there is an association with the human population. Among the East Asian-type CagA, 32 strains (53.3%) possessed ICE*Hptfs*. The ABBD type *cagA* strain did not contain any ICE*Hptfs* elements. The ABB type *cagA* containing ICE*Hptfs* were 4 of 11 strains (36.3%) and seemed to be equally distributed.

CagL Hypervariable Motif (CagLHM) had a close relationship with the geographical origin of *H. pylori*, as recently reported^25^. We evaluated the CagLHM and found 8 unique motifs. The predominant motifs were YEIGK, DEIGK and DKMGE (38.6%, 14.8% and 13.8%) (Fig. 1C). Interestingly we also found a novel motif DKMGK and this motif mostly was observed from *H. pylori* isolated from Samosir Island (Supplementary Table 1). This novel motif strains almost exclusively (91%) possessed ICE*Hptfs* elements as exclusive as the DKMGE motif strains (92.8%) (Fig. 1D).

### The *cag* PAI and histological findings

**Figure 2:**
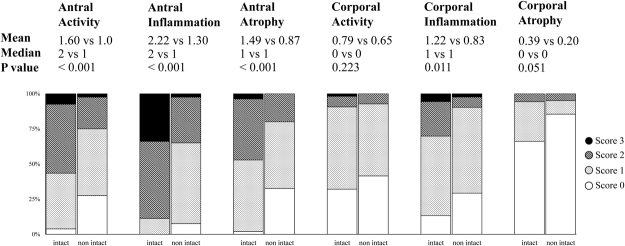
Association of *cag* PAI intactness and the histological findings. Patients infected with the intact *cag* PAI (n = 53) showed significantly higher antral activity, inflammation and atrophy as well as corporal inflammation than the non-intact counterpart (n = 40).

Comparison between histological findings and *cag* PAI intactness showed that patients infected with intact *cag* PAI had higher both corporal and antral inflammation than those with non-intact *cag* PAI (P = 0.011 and P < 0.001, respectively). Patients infected with intact *cag* PAI strains also showed higher activity and atrophy in the antrum than those with non-intact *cag* PAI strains (P < 0.001) (Fig. 2). Patients infected with intact *cag* PAI strains had significantly higher risk of antral activity, inflammation and atrophy and corporal inflammation and atrophy after adjusted with age and sex (Supplementary Table 3).

### The ICE*Hptfs* and histological findings

**Figure 3:**
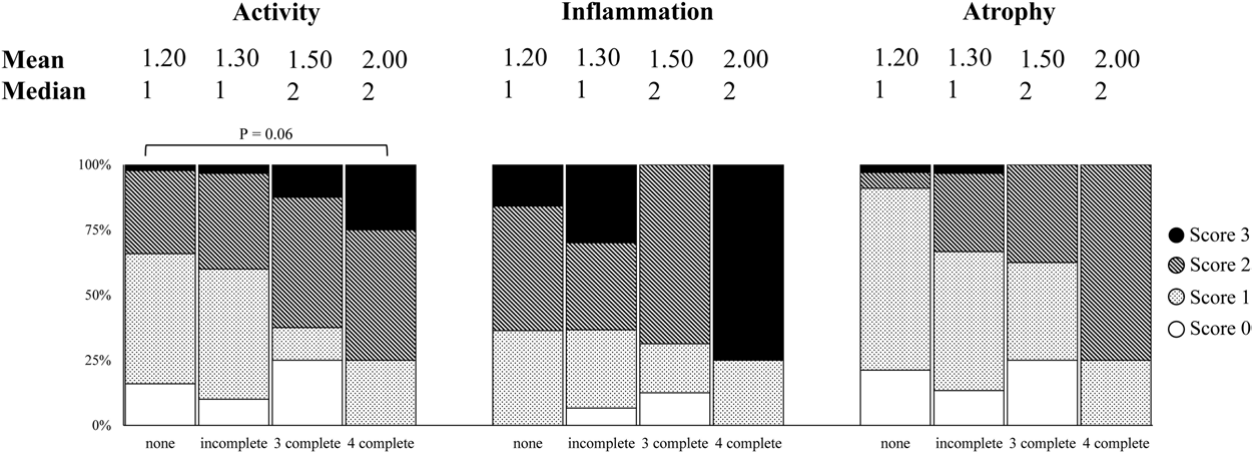
Association of ICE*Hptfs* and histological findings in antrum. Patients infected with the complete ICE*Hptfs4b* (n = 4) tended to have higher antral activity than antral activity than the ICE*Hptfs* negative (n = 44).

Histological examination showed the patients infected with strains possessing ICE*Hptfs* elements (either complete or incomplete) had significantly lower antral *H. pylori* density than those without (P = 0.039) (Table 3). As for the comparison between complete and incomplete ICE*Hptfs*(s), histological findings did not show any significant association; however, the patients infected with strains possessing complete ICE*Hptfs4b* tended to have higher activity in the antrum than those possessing ICE*Hptfs*-negative strains (P = 0.06) (Fig. 3).

**Table 3:** Association of ICE*Hptfs* status and histology score.

Histology score	ICE*Hptfs* positive (mean [median]) n = 47	ICE*Hptfs* negative (mean [median]) n = 56
**Antrum**		
Activity	1.44 [1.5]	1.20 [1]
Inflammation	1.82 [2]	1.79 [2]
Atrophy	1.24 [1]	1.18 [1]
Density*	1.19 [1]	1.53 [2]
**Corpus**		
Activity	0.72 [1]	0.73 [1]
Inflammation	1.04 [1]	1.04 [1]
Atrophy	0.32 [0]	0.28 [0]
Density	1.13 [1]	1.27 [1]

(*) P = 0.039, Mann-Whitney U test.

### Combination of the ICE*Hptfs*, *cag* PAI and histological findings

We classified *H. pylori* strains according to both the *cag* PAI intactness and status of ICE*Hptfs*, and examined the association between the combined classification and the histological scores. The patients infected with the strains possessing the combination of intact *cag* PAI-ICE*Hptfs*-positive strains had significantly higher antral activity compared to those with non-intact *cag* PAI-ICE*Hptfs*-negative as well as the non-intact *cag* PAI-ICE*Hptfs*-positive strains (P = 0.002 and P = 0.002, respectively) (Table 4). However, patients infected with the intact *cag* PAI-ICE*Hptfs*-negative strains did not show difference of antral activity compared to those with non-intact *cag* PAI-ICE*Hptfs*-negative strains (P = 0.103), suggesting that intact *cag* PAI virulence for inducing acute inflammation in the antrum is dependent on the status of ICE*Hptfs*. In addition, patients infected with intact *cag* PAI-ICE*Hptfs*-positive strains showed significantly higher antral inflammation and atrophy compared to those with non-intact *cag* PAI-ICE*Hptfs*-negative strains (P < 0.001 and P < 0.001). Corporal inflammation was also significantly higher in patients infected with intact *cag* PAI–ICE*Hptfs*-positive strains than those with non-intact *cag* PAI-ICE*Hptfs*-positive strains (P = 0.047). In addition, we also classified the strains based on the intactness of *cag* PAI and type of ICE*Hptfs*, then evaluated association with the histological scores. Despite the number of the samples being small for strains possessing intact *cag* PAI-complete ICE*Hptfs4b* (n = 3), we found that patients infected with these strains had higher antral activity and inflammation compared to those with non-intact *cag* PAI-incomplete ICE*Hptfs* strains (P = 0.024 and P = 0.009, respectively) (Table 4).

**Table 4:** Association of ICE*Hptfs* and *cag* PAI status and histology score.

*cag* PAI and ICE*Hptfs*	n	Antrum (Mean [Median])				Body (Mean [Median])			
		Activity	Inflammation	Atrophy	Density	Activity	Inflammation	Atrophy	Density
***cag*** **PAI and ICE** ***Hptfs*** **status**									
Intact *cag* PAI-ICE*Hptfs* +	34	1.75 [2]	2.25 [2]	1.47 [1]	1.45 [1]	0.75 [1]	1.13 [1]	0.38 [0]	1.25 [1]
Intact *cag* PAI-ICE*Hptfs*−	23	1.38 [1]	2.19 [2]	1.52 [1]	1.62 [2]	0.86 [1]	1.38 [1]	0.43 [0]	1.38 [1]
Non Intact *cag* PAI-ICE*Hptfs* +	21	0.94 [1]	1.13 [1]	0.94 [1]	0.76 [1]	0.69 [1]	0.94 [1]	0.25 [0]	1.00 [1]
Non Intact *cag* PAI-ICE*Hptfs*−	24	1.04 [1]	1.43 [1]	0.87 [1]	1.46 [1.5]	0.63 [1]	0.75 [1]	0.17 [0]	1.17 [1]
***cag*** **PAI and type of TFSS**									
Intact *cag* PAI-incomplete TFSS	21	1.58 [2]	2.37 [2]	1.47 [1]	1.60 [1.5]	0.53 [1]	1.11 [1]	0.32 [0]	1.21 [1]
Intact *cag* PAI-complete TFSS3	10	1.90 [2]	1.80 [2]	1.30 [1]	1.20 [1]	1.10 [1]	1.30 [1]	0.60 [0]	1.40 [1]
Intact *cag* PAI-complete TFSS4b	3	2.33 [2]	3.00 [3]	2.00 [2]	1.00 [1]	1.00 [1]	0.67 [0]	0.00 [0]	1.00 [1]
Non intact *cag* PAI-incomplete TFSS	10	1.00 [1]	1.11 [1]	1.00 [1]	1.25 [1]	0.88 [1]	1.13 [1]	0.50 [0]	1.25 [1]
Non intact *cag* PAI-complete TFSS3	9	0.83 [0.5]	1.17 [1.5]	0.83 [0.5]	0.75 [0]	0.43 [0]	0.71 [1]	0.00 [0]	0.83 [0.5]
Non intact *cag* PAI-complete TFSS4b^‡^	1	1	1	1	0	1	1	1	1

Abbreviations: PAI, Pathogenicity Island, TFSS, type IV secretion system ^‡^Number samples of this group only one sample. So we cannot calculate the mean and median.

## Discussion

This is the first study to evaluate the pathogenic role of ICE*Hptfs* in combination with *cag* PAI at a population level. We examined the prevalence of ICE*Hptfs* and *cag* PAI using high throughput sequencing. The previous genomic comparison showed that the ICE*Hptfs* has high prevalence (86.7%) in 45 strains worldwide^16^. We applied the same methods to determine the prevalence of ICE*Hptfs* among Indonesian strains, which showed a lower prevalence of ICE*Hptfs* (53.4%). In general, ICEs were transferred between genomes using conjugative HGT. This different prevalence might be due to observation only performed in one country compared to the worldwide observation. In addition, the distribution of ICE*Hptfs* had a significant association with ethnic groups in Indonesia, suggesting the prevalence and type of ICE*Hptfs* had an association with geographical origin. Some particular CagA genotypes strains did not possess any ICE*Hptfs*, especially the strains isolated from Merauke city. All our strains isolated from Merauke city were assigned as hpSahul (data not shown) and other strains deposited in the GenBank belonging to hpSahul, PNG84A and ausaBRJ05^26^, also did not contain ICE*Hptfs*, strongly supporting this association.

The *cag* PAI was transferred into *H. pylori* far prior human migrate from Africa 60 kya^27^ and interestingly, the *cag* PAI still can be observed in all the *H. pylori* populations after long period of human migration and shows the same evolution pattern as the house-keeping genes^27^. This suggests the importance of *cag* PAI towards the host colonization process. Our study showed almost all the Indonesian strains (98%) contained *cag* PAI, supporting its importance. In addition to the *cag* PAI, CagLHM may also help to discriminate geographical origin^25^. Our study showed that the predominant CagLHM in Indonesia were specifically observed in the East/Southeast Asia/Australasia groups, as previously reported^25^. We also found a new motif of CagLHM which showed as high prevalence of ICE*Hptfs* as the DKMGE motif. DKMGE is believed to be the progenitor of the CagLHM motif^25^, and since the observed motifs only differed on the residue 62 (E62K), this observation suggests the new motifs were directly derived from the progenitor.

The *cag* PAI was originally designated as a TFSS, which mainly has a function to translocate CagA protein into host cell cytoplasm^28,29^. However, the virulence of this island dependent to the intactness of this island, therefore it may successfully inject the CagA protein^12^. Our previous study in Vietnam^30^ classified the intactness of *cag* PAI based on the existence of the gene using the PCR method. Our current results showed similarly that the intact *cag* PAI has more severe histological score than the non-intact *cag* PAI. However, the previous criterion evaluated the intactness of *cag* PAI only based on the presence or absence of the member genes and the resulting high prevalence of intact *cag* PAI, which may blur the association with histological scores. On the other hand, the evaluation of *cag* PAI sequences may give us a significant association with the histological scores. In addition, it may also discriminate the cluster of *cag* PAI genotype (East-Asian type and Western type cluster), of which the East-Asian type may bind stronger to the SHP-2 receptor^11^. Therefore, we recommend the criteria to evaluate intactness of *cag* PAI also considering the functional status of the genes, as a more reliable method to predict clinical outcome.

Although strains with ICE*Hptfs* showed significantly lower *H. pylori* density, there was an association between a complete TFSS and the histological scores. It was reported that strains with a complete cluster of *dupA*, the VirB4 homologue of ICE*Hptfs4b*, lead to a higher risk of developing duodenal ulcers than those with incomplete *dupA* clusters or *dupA* negative strains^31^. Our data also showed the same tendency, even with a lower density in the antrum, suggesting this region has a more significant association to the *H. pylori* virulence, resulting in a higher active inflammation rather than attachment to the gastric mucosa.

In addition, we combined the status and type of ICE*Hptfs* with the *cag* PAI intactness. Our data showed patients infected with intact *cag* PAI-ICE*Hptfs*-positive strains had higher antral activity than those with non-intact *cag* PAI-ICE*Hptfs*-negative strains. However, patients infected with the intact *cag* PAI-ICE*Hptfs*-negative strains did not show difference of antral activity compared to those with non-intact *cag* PAI-ICE*Hptfs*-negative strains. These data suggest that the *cag* PAI and ICE*Hptfs* were dependent each other to induce higher antral activity. The TFSS can be divided into three groups according to their function^32^. The first group is the conjugation system, translocating single-stranded DNA substrates to recipient cells in a contact-dependent manner, resulting in the adaptation of bacteria to environmental changes. The second group is the effector translocation system, delivering protein directly into eukaryotic cells. The third group is the DNA uptake mediators, which uptake or release DNA or protein substrates extracellularly, independently of contact with another cell^33^. Since there was an evidence that the ICE*Hptfs* was a genetic mobile element which was transferred in the conjugation manner^16,34^, we assumed the function of ICE*Hptfs* in the pathogenesis of *H. pylori* infection was belongs to the conjugation group^33^, suggesting the ICE*Hptfs* might supporting the *cag* PAI to induce more severe clinical outcome.

Although we could not make strong conclusions due to a small sample size, particularly in the certain ICE*Hptfs* groups, this study gives us new information about the distribution and clinical association of this relatively new TFSS in *H. pylori*. In addition, since there have not been many biological and structure evidences of this particular system, further study is needed to better understand the role of the TFSS in colonization by *H. pylori*.

## Conclusion

In conclusion, our data showed a high prevalence of *cag* PAI in Indonesia, half of which were complete. Criteria determining intactness of *cag* PAI based on the gene functionality is more reliable to evaluate the influence of *H. pylori* on gastric mucosal status. The ICE*Hptfs* strains tended to induce more active inflammation in the antrum even with a lower density of bacteria. In combination, it was shown that patients infected with intact *cag* PAI-ICE*Hptfs*-positive strains had more severe inflammation than those with non-intact *cag* PAI-ICE*Hptfs*-negative strains, suggesting possibility a mutual correlation between these TFSS(s).

## Materials and Methods

### Samples and DNA sequencing

We performed endoscopic examination on 1072 dyspeptic patients in 17 cities in Indonesia from August 2012 to August 2016. We excluded patients with partial/total gastrectomy, non-fasted patients and those with contraindication for upper endoscopy. Written informed consent was obtained from all patients and the study protocol was approved by the ethics committees of Dr. Soetomo Teaching Hospital (Surabaya, Indonesia), Dr. Cipto Mangunkusumo Teaching Hospital (Jakarta, Indonesia), Dr. Wahidin Sudirohusodo Teching Hospital (Makassar, Indonesia) and Oita University Faculty of Medicine (Yufu, Japan). We declare that all procedures contributing to this work comply with the ethical standards of the relevant national and institutional committees on human experimentation and with the Helsinki Declaration of 1975, as revised in 2008 and 2013. We used antral gastric biopsy to isolate *H. pylori* as previously described^24^, resulting in 103 cultured isolates, including 75 isolates from our previous study^24^.

DNA extraction was performed using QIAamp DNA Mini Kit (QIAGEN, Valencia, CA, USA) following the manufacturer’s instructions. Whole genome sequencing was performed using a high throughput next generation sequencer; Illumina Hiseq. 2000 and Miseq as per the list in Supplementary Table 2. Briefly, high-quality genomic DNA was used, then was prepared using dual-indexed Nextera XT Illumina libraries and subjected to cluster generation and paired-end sequencing (2 × 300 bp) for Miseq and (2 × 150 bp) for Hiseq. We performed the quality control and de novo assembly prior the reference mapping to obtain the coverage and to select the result which may be used for further analysis using CLC Genomic Workbench v. 7.04, a commercial software (Qiagen Inc., Redwood, California, USA). The coverage we obtained was between 81–400 folds in each genome (Supplementary Table 1). The threshold for further analysis in this study, we use Q30 > 80% as recommended by Illumina and the average coverage more than 80 folds as had been described previously^35^.

### Analysis of ICE and other virulence genes

Identification of the ICE*Hptfs-*type was performed by using a reference mapping method. Short-read outputs were mapped to the corresponding reference sequences consisting of ICE*Hptfs3* (strain Gambia94/24), ICE*Hptfs4a* (strain P12) ICE*Hptfs4b* (strain G27) ICE*Hptfs4c* (strain SouthAfrica7) using CLC Genomic Workbench v. 7.04, a commercial software (Qiagen Inc., Redwood, California, USA) as described previously^16^. The unmapped reads then also assembled by using *de novo* assembly by the CLC Genomic Workbench. The ICE genes were identified by BLAST search (http://blast.ncbi.nlm.nih.gov/Blast.cgi) from the mapped reads. The *cag* PAI were identified using BLAST method and the query from strain 26995^8,27^. The functional status of the each gene was evaluated by visual inspection using MEGA7^36^.

### Histological evaluation

All biopsy material for histological evaluation was fixed in 10% buffered formalin and embedded in paraffin. Serial sections were stained with hematoxylin and eosin as well as May-Giemsa stains. Gastric mucosa were evaluated based on the updated Sydney system^37^. Bacterial load was classified into four grades: 0, ‘normal’; 1, ‘mild’; 2, ‘moderate’; and 3, ‘marked’ according to the updated Sydney system^37^.

The degree of inflammation, neutrophil activity, atrophy and intestinal metaplasia were classified into four grades according to the updated Sydney system: 0, ‘normal’; 1, ‘mild’; 2, ‘moderate’; and 3, ‘marked’^37^. Immunohistochemistry for anti-*H. pylori* antibody was performed as previously described^38^.

### Statistical analysis

Data were analyzed using IBM SPSS Statistics, version 22 (IBM Corp., USA). Discrete variables were tested using the chi-square test; continuous variables were tested using Mann-Whitney *U* test. An ordinal regression model was used to calculate risk for developing higher histological score. A two-tailed P value < 0.05 was considered statistically significant.

### Availability of Nucleotide Sequences

The accession number for nucleotide sequences were deposited in DDBJ under accession number LC334483 – LC335589 and LC339076 – LC339479.

## Electronic supplementary material

Supplementary Information
