# Supplementary material for: Distribution and clinical associations of integrating conjugative elements and cag pathogenicity islands of Helicobacter pylori in Indonesia
Source: Sci Rep. 2018 Apr 17;8:6073. doi: 10.1038/s41598-018-24406-y (PMC5904169; doi:10.1038/s41598-018-24406-y)
Supplement: Supplementary file 1 — Supplementary Information [file 41598_2018_24406_MOESM1_ESM.pdf]

Distribution and clinical associations of integrating conjugative elements and *cag* pathogenicity islands of *Helicobacter pylori* in Indonesia

Langgeng Agung Waskito, Muhammad Miftahussurur, Maria Inge Lusida, Ari Fahrial Syam, Rumiko Suzuki, Phawinee Subsomwong, Tomohisa Uchida, Muhammad Hamdan, Nasronudin and Yoshio Yamaoka

Supplementary Material

Supp. Table 1. The detail information of strains and the NGS platform were used in this current study.

No	Strain	City of taking	NGS Platform	CagA	CagLHM	Q_{30} Percentage	Avg. Coverage	Reference Study
1	IND7	Surabaya	Hiseq (PE 2x150)	ABB	DTTGE	90.99	346.57	[1]
2	IND68	Surabaya	Miseq (PE 2x300)	ABD	DEMGK	88.23	264.51	[1]
3	IND69	Surabaya	Hiseq (PE 2x150)	ABD	DKIGK	91.31	408.21	[1]
4	IND71	Surabaya	Hiseq (PE 2x150)	ABD	DKIGK	88.53	504.26	[1]
5	IND79	Surabaya	Hiseq (PE 2x150)	ABD	NA	88.06	560.21	[1]
6	JAY1	Jayapura	Hiseq (PE 2x150)	ABB	YEIGK	94.39	554.35	[2]
7	JAY3	Jayapura	Hiseq (PE 2x150)	ABB	YEIGK	95.95	570.45	[2]
8	JAY4	Jayapura	Miseq (PE 2x300)	B	DEMGK	85.83	224.50	[2]
9	JAY6	Jayapura	Hiseq (PE 2x150)	ABB	DEMGK	96.31	560.85	[2]
10	JAY8	Jayapura	Hiseq (PE 2x150)	ABB	YEIGK	95.97	589.56	[2]
11	JAY12	Jayapura	Miseq (PE 2x300)	ABB	DEMGK	84.54	231.21	[2]
12	JAY15	Jayapura	Hiseq (PE 2x150)	ABB	YEIGK	96.36	489.67	[2]
13	JAY16	Jayapura	Hiseq (PE 2x150)	ABD	YEIGK	95.14	509.21	[2]
14	JKT9	Jakarta	Hiseq (PE 2x150)	ABD	DKMGK	96.49	502.21	[2]
15	MO17	Medan	Miseq (PE 2x300)	ABD	DKMGK	91.23	115.74	[2]
16	MO23	Medan	Miseq (PE 2x300)	ABD	DEIGK	80.59	142.34	[2]
17	MO27	Medan	Miseq (PE 2x300)	ABD	DKMGK	90.67	166.24	[2]
18	MN3	Medan	Miseq (PE 2x300)	ABD	DKIGK	80.76	91.58	[2]
19	MN10	Medan	Miseq (PE 2x300)	ABD	DKMGE	80.88	93.57	[2]
20	MN11	Medan	Miseq (PE 2x300)	ABD	DKMGK	80.44	85.96	[2]
21	SMS15	Samosir	Miseq (PE 2x300)	ABD	DKIGK	87.68	278.20	[2]
22	SMS19	Samosir	Miseq (PE 2x300)	ABD	DEIGK	80.59	260.45	[2]
23	SMS20	Samosir	Miseq (PE 2x300)	ABD	DEIGK	83.32	176.28	[2]

24	SMS22	Samosir	Miseq (PE 2x300)	ABD	DKMGK	82.27	164.28	2
25	SMS23	Samosir	Miseq (PE 2x300)	ABD	DKMGK	82.74	115.51	2
26	SMS24	Samosir	Miseq (PE 2x300)	ABD	DKIGK	81.06	264.07	2
27	SMS25	Samosir	Miseq (PE 2x300)	ABD	DKMGK	80.97	219.52	2
28	SMS28	Samosir	Miseq (PE 2x300)	ABD	DKMGK	80.80	169.54	2
29	SMS30	Samosir	Miseq (PE 2x300)	ABD	DKMGK	80.42	237.04	2
30	MKS31	Makasar	Miseq (PE 2x300)	ABC	DKMGE	81.19	87.65	2
31	MKS45	Makasar	Miseq (PE 2x300)	AABD	YEIGK	89.51	156.35	2
32	MKS47	Makasar	Miseq (PE 2x300)	ABC	YEIGK	80.68	85.35	2
33	MKS52	Makasar	Miseq (PE 2x300)	AABD	YEIGK	83.13	87.07	2
34	MKS55	Makasar	Miseq (PE 2x300)	ABC	NKIGQ	83.49	89.29	2
35	MKS56	Makasar	Miseq (PE 2x300)	ABCC	DKIGK	82.46	82.43	2
36	PTN63	Pontianak	Miseq (PE 2x300)	ABC	DKMGE	88.97	82.77	2
37	PTN75	Pontianak	Miseq (PE 2x300)	ABC	YEIGK	86.89	85.13	2
38	PTK20	Pontianak	Miseq (PE 2x300)	BC	DKMGE	89.57	191.58	2
39	PTK44	Pontianak	Miseq (PE 2x300)	ABD	DEIGK	89.45	128.24	2
40	PTK50	Pontianak	Miseq (PE 2x300)	ABD	DKMGE	91.01	99.51	2
41	MANADO5	Manado	Miseq (PE 2x300)	ABD	DKIGK	89.76	171.08	3
42	MANADO18	Manado	Miseq (PE 2x300)	ABD	DKMGK	90.18	136.04	3
43	MANADO20	Manado	Miseq (PE 2x300)	ABD	DKIGK	87.37	110.47	3
44	MANADO26	Manado	Miseq (PE 2x300)	ABD	DKIGK	86.67	151.85	3
45	MANADO28	Manado	Miseq (PE 2x300)	ABD	YEIGK	93.44	175.98	3
46	MANADO29	Manado	Miseq (PE 2x300)	ABD	DKIGK	93.63	90.53	3
47	MANADO31	Manado	Miseq (PE 2x300)	ABD	DEIGK	93.18	102.87	3
48	KPG2	Kupang	Miseq (PE 2x300)	ABD	DKMGE	82.79	156.23	3

49	KPG5	Kupang	Miseq (PE 2x300)	ABD	DEIGK	85.25	132.20	3
50	KPG6	Kupang	Miseq (PE 2x300)	ABC	DEIGK	83.27	120.20	3
51	KPG10	Kupang	Miseq (PE 2x300)	ABC	DEIGK	82.16	105.54	3
52	KPG11	Kupang	Miseq (PE 2x300)	ABD	DEIGK	80.20	196.38	3
53	KPG15	Kupang	Miseq (PE 2x300)	ABD	YEIGK	82.36	192.96	3
54	KPG23	Kupang	Miseq (PE 2x300)	BC	DKMGE	84.94	125.05	3
55	KPG26	Kupang	Miseq (PE 2x300)	BC	DKMGE	89.29	98.87	3
56	KPG28	Kupang	Miseq (PE 2x300)	BC	DKMGE	86.58	146.88	3
57	KPG29	Kupang	Miseq (PE 2x300)	ABD	DEIGK	81.55	144.70	3
58	KPG30	Kupang	Miseq (PE 2x300)	ABC	DKMGE	85.47	85.85	3
59	KPG33	Kupang	Miseq (PE 2x300)	none	NA	83.55	158.02	3
60	KPG34	Kupang	Miseq (PE 2x300)	BC	DKMGE	87.82	238.16	3
61	KPG35	Kupang	Miseq (PE 2x300)	ABD	DTTGE	85.30	240.73	3
62	KPG41	Bangli	Miseq (PE 2x300)	ABC	NKIGQ	85.57	112.69	3
63	KPG42	Bangli	Miseq (PE 2x300)	AABD	YEIGK	81.43	86.98	3
64	KPG47	Bangli	Miseq (PE 2x300)	ABC	DKIGK	85.50	142.12	3
65	KPG64	Bangli	Miseq (PE 2x300)	AAD	DTTGE	82.59	171.52	3
66	KPG73	Bangli	Miseq (PE 2x300)	ABC	DKIGK	88.07	134.33	3
67	KPG83	Bangli	Miseq (PE 2x300)	ABD	YEIGK	85.08	152.03	3
68	MEDAN18	Medan	Miseq (PE 2x300)	ABD	YEIGK	89.67	201.24	3
69	MEDAN31	Medan	Miseq (PE 2x300)	ABD	DKMGK	90.39	162.60	3
70	MEDAN32	Medan	Miseq (PE 2x300)	ABD	DEIGK	88.11	264.33	3
71	MEDAN33	Medan	Miseq (PE 2x300)	ABD	DEIGK	86.63	368.99	3
72	NIAS9	Nias	Miseq (PE 2x300)	ABD	YEIGK	84.92	123.05	this study
73	NIAS36	Samosir	Miseq (PE 2x300)	ABD	DKMGE	84.74	97.32	this study

74	NIAS37	Samosir	Miseq (PE 2x300)	ABD	DKMGE	85.43	95.96	this study
75	NIAS40	Samosir	Miseq (PE 2x300)	ABD	DEIGK	85.11	125.98	this study
76	NIAS49	Samosir	Miseq (PE 2x300)	ABD	YEIGK	83.54	163.37	this study
77	NIAS50	Samosir	Miseq (PE 2x300)	ABD	YEIGK	83.51	146.38	this study
78	NIAS56	Samosir	Miseq (PE 2x300)	ABBD	YEIGK	83.51	192.02	this study
79	NIAS67	Samosir	Miseq (PE 2x300)	ABD	YEIGK	85.69	223.14	this study
80	NIAS68	Samosir	Miseq (PE 2x300)	ABD	YEIGK	84.11	137.93	this study
81	NIAS73	Samosir	Miseq (PE 2x300)	ABBD	YEIGK	83.87	224.19	this study
82	NIAS75	Samosir	Miseq (PE 2x300)	ABBD	YEIGK	83.86	194.47	this study
83	PDG42	Palembang	Miseq (PE 2x300)	ABD	YEIGK	88.29	157.75	this study
84	SBY106	Surabaya	Miseq (PE 2x300)	ABD	DEIGK	80.06	84.06	[2]
85	SBY137	Surabaya	Miseq (PE 2x300)	ABD	DKMGE	86.49	104.09	[2]
86	SBY151	Surabaya	Miseq (PE 2x300)	ABC	NKIGQ	82.68	96.79	[2]
87	SBY192	Surabaya	Miseq (PE 2x300)	ABD	DEIGK	85.02	106.55	[2]
88	MER3	Merauke	Miseq (PE 2x300)	B	YEIGK	85.05	243.44	this study
89	MER5	Merauke	Miseq (PE 2x300)	B	YEIGK	84.41	219.32	this study
90	MER7	Merauke	Miseq (PE 2x300)	B	YEIGK	83.16	225.09	this study
91	MER8	Merauke	Miseq (PE 2x300)	B	YEIGK	86.05	269.32	this study
92	MER12	Merauke	Miseq (PE 2x300)	AB	YEIGK	85.70	191.03	this study
93	MER20	Merauke	Miseq (PE 2x300)	AB	YEIGK	88.24	145.23	this study
94	MER21	Merauke	Miseq (PE 2x300)	B	YEIGK	87.53	338.84	this study
95	MER27	Merauke	Miseq (PE 2x300)	B	YEIGK	80.84	185.99	this study
96	MER37	Merauke	Miseq (PE 2x300)	AB	YEIGK	86.56	304.88	this study
97	KOL56	Kolaka	Miseq (PE 2x300)	none	YEIGK	85.48	219.48	this study
98	KOL72	Kolaka	Miseq (PE 2x300)	ABC	YEIGK	86.86	196.53	this study

99	KOL79	Kolaka	Miseq (PE 2x300)	ABC	YEIGK	86.15	343.49	this study
100	KOL94	Kolaka	Miseq (PE 2x300)	ABC	YEIGK	83.82	256.40	this study
101	KOL96	Kolaka	Miseq (PE 2x300)	ABC	YEIGK	82.58	190.85	this study
102	KOL98	Kolaka	Miseq (PE 2x300)	AB	YEIGK	83.99	206.04	this study
103	KOL99	Kolaka	Miseq (PE 2x300)	ABC	YEIGK	81.86	279.25	this study

Abbreviations: NGS, Next-generation sequencing; CagLHM, CagL Hypervariable Motif.; Avg. Average

Table 2. The CagA genotype and the distribution of ICE*Hptfs*

CagA Genotype	Total	ICE*Hptfs*	ICE*Hptfs* Profiles (%)					
			**TFSS3**	**TFSS4a**	**TFSS4b**	**TFSS3-4a**	**TFSSS3-4b**	**TFSS3-4a/4b**
**Western type**	30	18 (60.0)	5 (27.8)	4 (22.2)	2 (11.1)	2 (11.1)	2 (11.1)	3 (16.7)
ABC	17	12 (70.6)	4 (33.3)	4 (33.3)	2 (16.7)	1 (8.3)	1 (8.3)	0 (0.0)
ABCC	1	0 (0.0)	0 (0.0)	0 (0.0)	0 (0.0)	0 (0.0)	0 (0.0)	0 (0.0)
BC	5	2 (40.0)	1 (50.0)	0 (0.0)	0 (0.0)	1 (50.0)	0 (0.0)	0 (0.0)
**EA type**	60	32 (53.3)	3 (9.4)	4 (12.5)	3 (9.4)	19 (59.4)	2 (6.3)	1 (3.1)
AABD	3	0 (0.0)	0 (0.0)	0 (0.0)	0 (0.0)	0 (0.0)	0 (0.0)	0 (0.0)
AAD	1	1 (100.0)	0 (0.0)	0 (0.0)	0 (0.0)	1 (100.0)	0 (0.0)	0 (0.0)
ABBD	3	0 (0.0)	0 (0.0)	0 (0.0)	0 (0.0)	0 (0.0)	0 (0.0)	0 (0.0)
ABD	53	31 (58.5)	3 (9.7)	4 (12.9)	3 (9.7)	18 (58.1)	2 (6.5)	1 (3.2)
**ABB type**	15	4 (36.4)	1 (25.0)	0 (0.0)	1 (25.0)	1 (25.0)	1 (25.0)	0 (0.0)
ABB	7	3 (42.8)	0 (0.0)	0 (0.0)	1 (33.3)	1 (33.3)	1 (33.3)	0 (0.0)
AB	4	1 (25)	1 (100)	0 (0.0)	0 (0.0)	0 (0.0)	0 (0.0)	0 (0.0)
B	7	0 (0.0)	0 (0.0)	0 (0.0)	0 (0.0)	0 (0.0)	0 (0.0)	0 (0.0)

Abbreviations: EA, East-Asian; TFSS, type IV secretion system

Table 3. The Calculated Odd Ratio of Gastric Mucosal Status between intact *cag*PAI infected patients and non-intact cag PAI infected patients

Histology Evaluation	OR*	95%CI	P value
Antral Activity	5.03	2.14-11.76	<0.001
Antral Inflammation	18.52	6.36-52.63	<0.001
Antral Atrophy	5.71	2.32-14.08	<0.001
Corporal Activity	1.63	0.72-3.70	0.239
Corporal Inflammation	3.23	1.36-7.63	0.008
Corporal Atrophy	2.90	1.02-8.19	0.045

Abbreviations: OR, Odd ratio; CI, Confidence interval.

*) Odd ratio was calculated using ordinal-regression model, considering the age and sex as the cofactors

Supplementary Figure 1

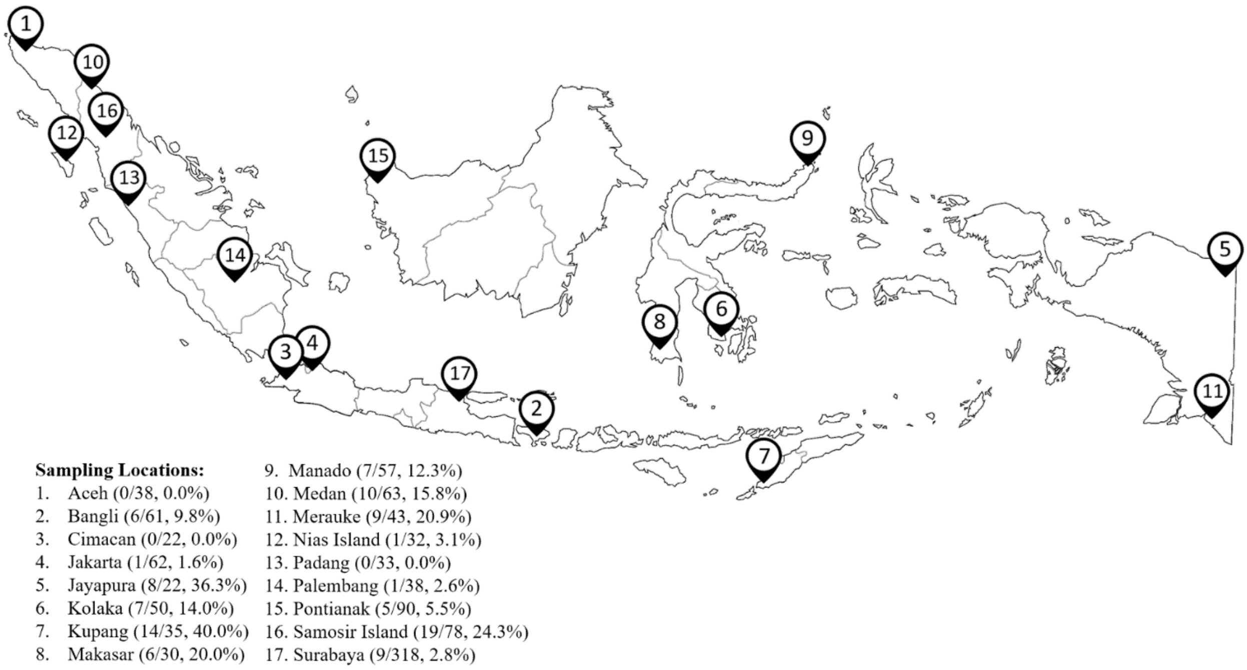

The Place for Taking Endoscopic Examination in Indonesia

We performed endoscopic examination to 1072 dyspeptic patients in the 17 cities in Indonesia. Among 1072 examined patients, we could culture 103 *H. pylori*.

Supplementary figure 2

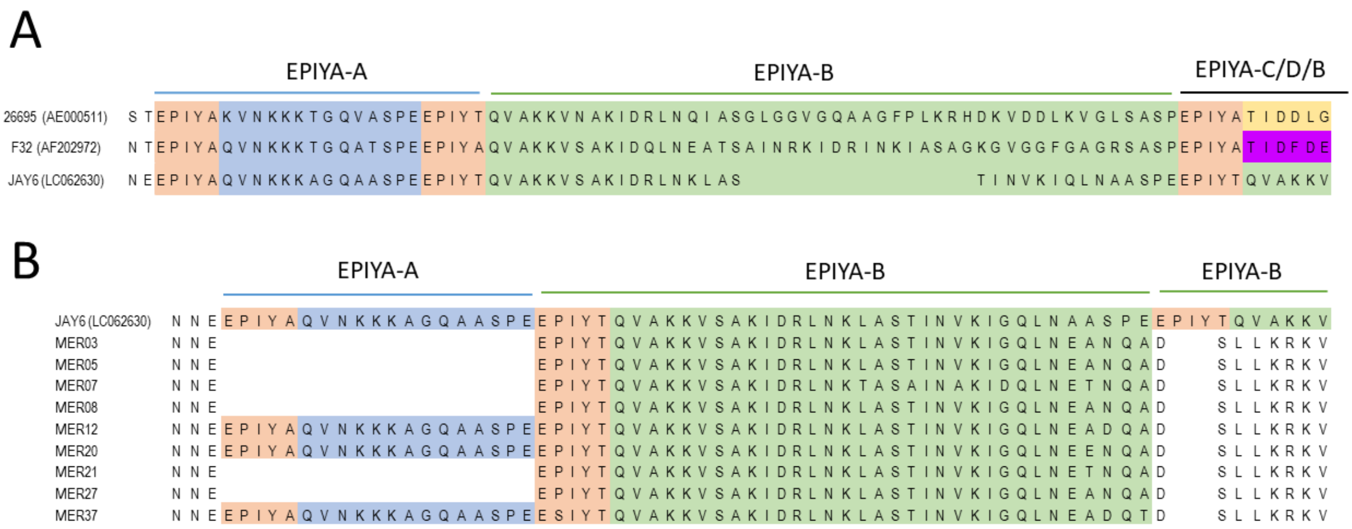

The genetic polymorphism on the CagA which may differentiate the EPIYA segments become EPIYA-A, EPIYA-B and EPIYA-C/D.

A) The polymorphism of CagA may different in each strain, dependent to the geographical origin of the isolates. Strains 26695 (AE000511) which isolated from England showed ABC type CagA (Western-type), whereas strain F32 (AF202972) showed ABD type CagA (East Asian-type). The unique type of CagA was also found in JAY6, isolated in Jayapura, Papua Island which had ABB type CagA.

B) The strains isolated from Merauke, Papua Island showed only had AB or B type of CagA. The B-segment of CagA was very similar to B-segment CagA from JAY6 which may classified as subtype of ABB type CagA.

Supplementary figure 3

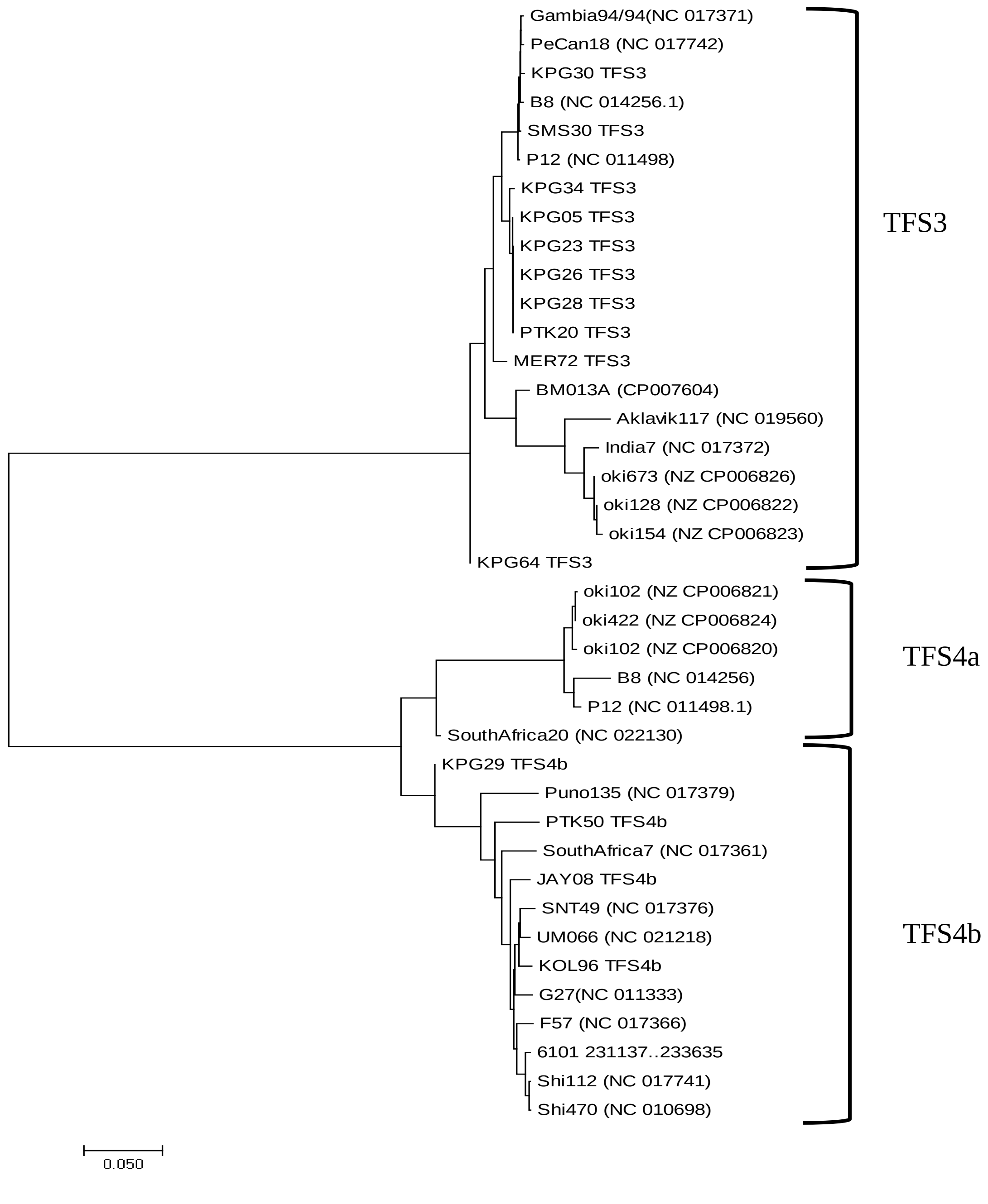

Neighbor-joining analysis of type IV secretion system gene sequences.

Phylogenetic tree calculated from concatenated *vir*B4, *vir*B9 and *vir*B10 ortholog sequences of all ICE*Hptfs3* and ICE*Hptfs4* island of Indonesian strains together with the whole genome available in Genbank, resulting a very distinctive between TFSS3 and TFSS4. Within the TFSS4, there is a subtype of TFSS4a and TFSS4b.

Supplementary References

1 Miftahussurur, M. *et al.* Identification of *Helicobacter pylori* infection in symptomatic patients in Surabaya, Indonesia, using five diagnostic tests. *Epidemiology and infection* **143**, 986-996, doi:10.1017/S095026881400154X (2015).

2 Miftahussurur, M. *et al.* *Helicobacter pylori* virulence genes in the five largest islands of Indonesia. *Gut pathogens* **7**, 26, doi:10.1186/s13099-015-0072-2 (2015).

3 Miftahussurur, M. *et al.* Surveillance of *Helicobacter pylori* Antibiotic Susceptibility in Indonesia: Different Resistance Types among Regions and with Novel Genetic Mutations. *PloS one* **11**, e0166199, doi:10.1371/journal.pone.0166199 (2016).
